# Noninvasive Assessment of Hepatic Steatosis in Living Liver Donors

**DOI:** 10.3390/diagnostics16050772

**Published:** 2026-03-04

**Authors:** Iman Al-Saleh, Hamad Alashgar, Ali Albenmousa, Ruba Alsaeed, Madiha Jamal

**Affiliations:** 1 Environmental Health Program, King Faisal Specialist Hospital and Research Centre, P.O. Box 3354, Riyadh 11211, Saudi Arabia; 2 Gastroenterology Section, Department of Medicine, King Faisal Specialist Hospital and Research Centre, P.O. Box 3354, Riyadh 11211, Saudi Arabia; 3 Transplant Endoscopy Unit, Liver and Small Bowel Health Centre, King Faisal Specialist Hospital and Research Centre, P.O. Box 3354, Riyadh 11211, Saudi Arabia; 4 School of Medicine, Alfaisal University, Riyadh 11533, Saudi Arabia

**Keywords:** hepatic steatosis, living liver donor, MRI-PDFF, transient elastography, controlled attenuation parameter (CAP), CT attenuation, FIB-4, APRI, noninvasive diagnostics, MASLD

## Abstract

**Background & Aims:** The accurate, noninvasive assessment of hepatic steatosis is essential in living liver donor evaluation, where disease prevalence is low, and donor safety is paramount. This study evaluated commonly used noninvasive diagnostic tools for detecting hepatic steatosis in a real-world donor screening setting. **Methods:** We analyzed 108 living liver donor candidates (18–53 years) with complete MRI, CT, transient elastography (FibroScan^®^), and biochemical data obtained during routine donor evaluation. Hepatic steatosis was defined as an MRI-proton density fat fraction (PDFF) ≥5%, which served as the noninvasive reference standard. Diagnostic performance metrics, receiver operating characteristic (ROC) analyses, and correlations with serum fibrosis indices (FIB-4 and APRI) were assessed. **Results:** MRI-PDFF identified hepatic steatosis in 21 donors (19.4%). Controlled attenuation parameter (CAP), measured by transient elastography, demonstrated high sensitivity (90.5%) and negative predictive value (97.1%), supporting its role as a rule-out screening tool. CT showed excellent specificity (97.7%) but lower sensitivity (61.9%), consistent with a confirmatory role when MRI is unavailable. Serum fibrosis indices were generally low and did not correlate strongly with imaging-based steatosis. **Conclusions:** In the low-prevalence setting of living liver donor evaluation, CAP-based transient elastography provides effective noninvasive screening for hepatic steatosis, while MRI-PDFF serves as a confirmatory reference when indicated. These findings support a stepwise, clinically practical diagnostic approach that prioritizes donor safety and workflow efficiency.

## 1. Introduction

Metabolic dysfunction-associated steatotic liver disease (MASLD), previously termed non-alcoholic fatty liver disease (NAFLD), is the most prevalent chronic liver condition worldwide, affecting approximately 25% of the global population [1,2]. Despite its high prevalence, MASLD often remains undiagnosed due to its asymptomatic nature and the limited accessibility of definitive diagnostic tools. Imaging therefore plays a critical role in detection, particularly in specific clinical contexts such as living liver donor evaluation, where the accurate exclusion of hepatic steatosis is essential for donor safety.

Although ultrasonography is widely available and demonstrates good diagnostic accuracy for detecting hepatic steatosis, its performance may be operator-dependent and less reproducible for precise fat quantification in low-prevalence screening settings such as living liver donor evaluation [3]. Magnetic resonance imaging (MRI), particularly when utilizing proton density fat fraction (PDFF), offers superior diagnostic accuracy and is currently regarded as the noninvasive reference standard for quantitative hepatic fat assessment, but is limited by cost and availability [4]. In this setting, MRI-PDFF provides a reproducible, noninvasive reference method, especially valuable when liver biopsy is neither clinically indicated nor ethically appropriate. Transient elastography, performed using FibroScan^®^, quantifies hepatic fat through the controlled attenuation parameter (CAP) and has been increasingly incorporated into donor evaluation protocols because of its reproducibility and ability to detect early disease [5,6]. Computed tomography (CT), while occasionally used in transplant evaluations, is less sensitive and lacks the quantitative precision of MRI [7]. Nonetheless, CT may retain clinical value because of its high specificity, and studies such as those by Kim et al. [8] have reported that CT can detect even low-grade hepatic steatosis in asymptomatic individuals during health screenings.

Beyond steatosis detection, comprehensive donor assessment also requires evaluating liver fibrosis, which has important implications for donor safety and long-term liver function. Liver biopsy has historically served as the reference standard for assessing hepatic steatosis and fibrosis; however, in living donor candidates it is not routinely justified because it exposes otherwise healthy individuals to procedural risks (including pain, bleeding, and rare serious complications), in addition to potential sampling variability, thereby limiting its routine applicability in this population [4,9]. In this context, noninvasive serum-based indices offer a practical alternative for estimating fibrosis risk. Noninvasive serum-based indices, such as the Fibrosis-4 (FIB-4) score and the aspartate aminotransferase-to-platelet ratio index (APRI), are widely validated tools for estimating liver fibrosis and cirrhosis [10,11]. They complement imaging-based modalities by providing biochemical insight into hepatic injury and fibrotic progression [12,13], although they are not designed to directly quantify hepatic fat content.

Accurate, noninvasive evaluation of both hepatic steatosis and fibrosis is essential in living donor assessment, where minimizing risk and avoiding unnecessary invasive procedures are central priorities. Unlike typical MASLD populations, potential living liver donors represent a low-prevalence, high-stakes clinical setting in which negative predictive value and workflow efficiency are particularly important. Despite the availability of multiple imaging tools, few studies have directly compared CAP-based transient elastography, CT, and serum fibrosis markers with MRI-PDFF within the same cohort of healthy living liver donors. Moreover, MRI-PDFF, which has been technically validated against MR spectroscopy, provides a reproducible and quantitative reference method for hepatic fat assessment [4] and has been widely adopted as a reference standard in comparative studies of noninvasive steatosis biomarkers [14]. To address this gap, the diagnostic performance of CAP-based transient elastography and CT was evaluated using MRI-PDFF as the reference standard, and the relationship between serum indices (FIB-4, APRI) and imaging findings in this donor population was examined.

## 2. Methods

### 2.1. Study Design and Participants

This retrospective cross-sectional study of a living liver donor cohort was conducted at the King Faisal Specialist Hospital and Research Centre (KFSH&RC), Riyadh, Saudi Arabia. It included healthy liver donors evaluated between 2023 and 2025. The study was designed to assess the real-world diagnostic performance of routinely used noninvasive tools within a standardized donor screening workflow. The study was approved by KFSH&RC Research Ethics Committee (RAC #2220021; approval date: 30 November 2022) and was conducted in accordance with the Declaration of Helsinki (1975, revised 2013) and institutional ethical standards.

A total of 176 donor records were reviewed. Of these, 108 donors had complete imaging and biochemical data and were included in the final analysis. Donors were excluded if imaging was incomplete, technically inadequate, or performed outside the standard donor evaluation protocol. All participants were evaluated as part of a standardized living liver donor assessment protocol and were considered clinically healthy at the time of imaging. Donors with known chronic liver disease, viral hepatitis, significant alcohol use, or other contraindications to donation were excluded during routine clinical screening. Baseline metabolic parameters and liver biochemistry were reviewed as part of the donor eligibility assessment. No participants had clinically suspected iron overload or advanced metabolic disease at the time of evaluation. As this was a retrospective study, subclinical conditions that may influence fat quantification could not be entirely excluded.

### 2.2. Imaging Modalities and Steatosis Assessment

Imaging data were extracted retrospectively from clinical records and assessed as follows. MRI, CT, and transient elastography (FibroScan^®^) examinations were performed within the standard donor evaluation window (typically within 1–2 weeks), thereby minimizing temporal variability across modalities and reflecting routine clinical sequencing.

• MRI: Hepatic steatosis was assessed using proton density fat fraction (PDFF) sequences, and all diagnoses were confirmed through clinical radiology reports. MRI-PDFF was used as the noninvasive reference standard for this study, consistent with its established validation against MR spectroscopy and histology in the prior literature. Imaging was performed using a 1.5T Siemens AERA (Siemens Healthineers, Erlangen, Germany) system. MRI examinations were acquired using a vendor-provided multi-echo spoiled gradient-echo sequence for PDFF estimation. PDFF maps were generated automatically by the scanner software using standard multi-echo acquisition and vendor-implemented correction algorithms designed to minimize known sources of bias in fat quantification, in accordance with routine clinical practice rather than research-specific post-processing pipelines. Whole-liver PDFF measurements were derived from regions of interest placed in representative liver segments, avoiding major vessels and bile ducts. All imaging examinations were acquired, interpreted, and reported as part of routine clinical care by board-certified radiologists, and no additional image reprocessing or segmentation was undertaken for research purposes, consistent with the study’s focus on real-world diagnostic performance.
• CT: Non-contrast CT scans were evaluated for hepatic attenuation and radiological evidence of steatosis. Only examinations with complete liver coverage were included. Scans were acquired using a Siemens SOMATOM Force (384-slice) CT scanner (Siemens Healthineers, Erlangen, Germany). CT images were obtained during a single breath-hold using standard non-contrast donor evaluation protocols. Hepatic attenuation measurements were derived from regions of interest placed in the right hepatic lobe, avoiding vascular structures and focal lesions, as documented in the original clinical radiology reports.
• Transient elastography (FibroScan^®^): Hepatic fat content was measured using the controlled attenuation parameter (CAP) obtained through transient elastography. A CAP cutoff of ≥248 dB/m was used to define steatosis [15]. Measurements were performed using the FibroScan 502 Touch device (Echosens, Paris, France). Operators performing transient elastography using FibroScan^®^ were unaware of MRI and CT results at the time of measurement and followed manufacturer recommendations. Appropriate probe selection was based on the patient’s body habitus. Measurements were considered reliable when at least ten valid acquisitions were obtained, and median values were used for analysis.

Radiologists interpreting the CT images and operators performing CAP-based transient elastography examinations were blinded to the MRI findings to minimize observer bias. For MRI and CT, hepatic fat assessment was based on region-of-interest (ROI) measurements rather than full volumetric segmentation. ROIs were placed in representative liver segments, avoiding major vessels, bile ducts, and focal lesions, consistent with routine clinical reporting practice. Measurements, therefore, reflected representative sampling, commonly used in donor screening, rather than exhaustive whole-liver quantification. Imaging assessments were performed as part of standard clinical donor evaluation and interpreted by experienced radiologists.

Formal assessment of hepatic fat heterogeneity across individual liver segments was not performed. Instead, PDFF and CT attenuation measurements were based on representative region-of-interest sampling in commonly evaluated liver segments, in line with routine clinical donor assessment protocols. This approach reflects standard practice in living donor screening, where the primary objective is exclusion of clinically significant steatosis rather than detailed characterization of regional fat distribution.

### 2.3. Serum Fibrosis Indices

In addition to imaging assessments, two noninvasive serum-based indices—Fibrosis-4 (FIB-4) and the Aspartate Aminotransferase-to-Platelet Ratio Index (APRI)—were calculated for each donor to estimate liver fibrosis and potential disease severity. These indices were included to contextualize fibrosis risk and were not intended to assess hepatic steatosis.

• FIB-4 index was calculated using the following formula:

FIB-4 = (Age [years] × AST [U/L])/(Platelet count [10^9^/L] × √ALT [U/L])

Values were categorized as: <1.3 (low risk), 1.3–2.67 (indeterminate), and >2.67 (high risk) for advanced fibrosis [16].

• APRI index was calculated using the formula:

APRI = (AST [U/L]/ULN AST [45 U/L])/Platelet count [10^9^/L] × 100

APRI values were interpreted as: <0.5 (low risk), 0.5–1.5 (indeterminate), and >1.5 (high risk) for significant fibrosis [17].

Both indices were derived from laboratory parameters obtained on the same day as the imaging, ensuring temporal consistency. All analyses were conducted at the Clinical Biochemistry/Hematology, Pathology, and Laboratory Medicine Department of KFSH&RC as part of the donor assessment.

### 2.4. Statistical Analysis

The diagnostic performance of CT and CAP-based transient elastography for detecting hepatic steatosis was evaluated using MRI-PDFF as the reference standard. Sensitivity, specificity, positive predictive value (PPV), negative predictive value (NPV), and their corresponding 95% confidence intervals (95% CI) were calculated. Given the low-prevalence donor setting, particular emphasis was placed on negative predictive value as a clinically relevant screening metric.

Receiver operating characteristic (ROC) curves were generated to assess overall diagnostic accuracy, and the area under the curve (AUC) with 95% CI was computed for each modality.

Group comparisons of serum fibrosis indices (FIB-4 and APRI) across binary imaging classifications (MRI-defined steatosis, CT-defined steatosis, and CAP ≥ 248 dB/m) were performed using the Mann–Whitney U test. Correlation analyses were limited to continuous serum indices (FIB-4 and APRI) and conducted using Spearman’s rank correlation coefficient (ρ).

All statistical analyses were conducted using IBM SPSS Statistics for Windows, Version 26.0 (IBM Corp., Armonk, NY, USA). A two-tailed *p*-value < 0.05 was considered statistically significant.

## 3. Results

### 3.1. Diagnostic Accuracy of Imaging Modalities

Among the 176 donors screened, 108 (aged 18–53 years; 75 males and 33 females) had complete MRI, CT, transient elastography (FibroScan^®^), and biochemical data and were included in the final donor cohort. MRI identified steatosis in 21 donors (19.4%), a prevalence consistent with the low disease burden typical of living liver donor screening. CAP-based transient elastography detected most MRI-defined steatosis cases, achieving a sensitivity of 90.5% and an NPV of 97.1%. A normal CAP value therefore reliably excluded steatosis in this cohort, supporting its use as a rule-out screening modality. CT showed a different pattern: although it missed several MRI-positive cases, it rarely generated false positives, resulting in high specificity (97.7%) and a PPV of 86.7%. These complementary performance characteristics highlight differing clinical roles for the two modalities. These results are summarized in Table 1 and Table 2.

**Figure 1:**
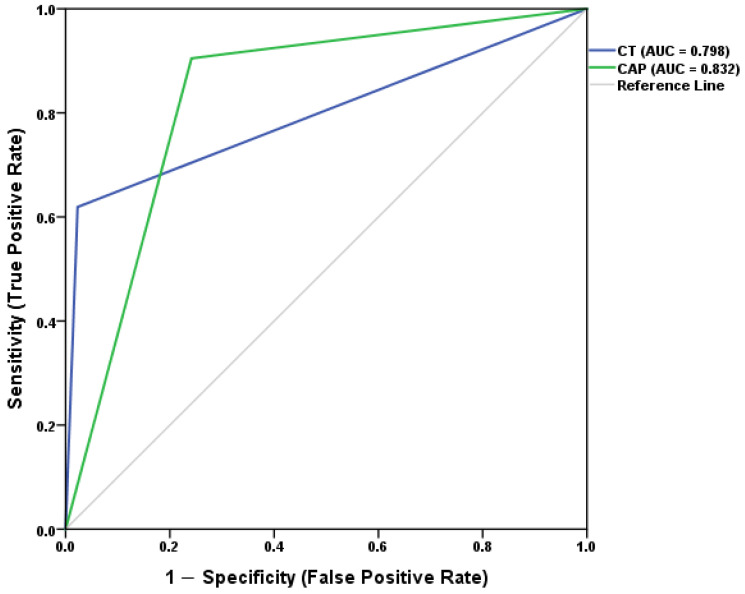
Receiver operating characteristic (ROC) curves for CT and CAP-based transient elastography (CAP ≥ 248 dB/m) compared with MRI-PDFF as the reference standard. CAP-based transient elastography achieved a higher area under the curve (AUC = 0.832; 95% CI, 0.74–0.924) than CT (AUC = 0.798; 95% CI, 0.667–0.929), indicating superior diagnostic accuracy for detecting hepatic steatosis at the higher CAP threshold.

Receiver operating characteristic (ROC) analysis (Figure 1) demonstrated an area under the curve (AUC) of 0.832 for CAP-based transient elastography and 0.798 for CT. The modest difference in AUC reflects trade-offs between sensitivity and specificity rather than a clear superiority of one modality across all diagnostic scenarios.

### 3.2. Serum Fibrosis Indices (FIB-4 and APRI)

The mean FIB-4 score was 0.65 ± 0.60, and the mean APRI score was 0.20 ± 0.17. Most donors were classified as low risk according to both indices, with 96.2% categorized as low risk by FIB-4 and 97.2% by APRI. These findings are consistent with the donor population’s clinically healthy status. Only a small proportion of donors fell into the intermediate- or high-risk categories (Table 3).

### 3.3. Associations Between Imaging and Serum Indices

Serum indices were compared across imaging categories. FIB-4 showed no meaningful differences between donors with and without MRI-, CT-, or CAP-defined steatosis. APRI was somewhat higher in the MRI-positive group, but not in the CT or CAP groups. Although these biochemical differences were observable, they remained within normal ranges for healthy adults and were not clinically significant.

FIB-4 and APRI demonstrated a moderate correlation (r = 0.55). Neither index showed a significant association with imaging-based steatosis measures, underscoring their primary role as fibrosis risk indicators rather than hepatic fat quantification tools.

## 4. Discussion

### 4.1. Comparative Diagnostic Performance and Clinical Implications

In this cohort of living liver donors, CAP-based transient elastography demonstrated strong rule-out performance as an initial screening tool. A normal CAP result reliably excludes hepatic steatosis, with important practical implications for donor evaluation workflows. This finding suggests that CAP-based transient elastography may reduce the need for additional imaging in a substantial proportion of donor candidates.

From a practical perspective, CAP-based transient elastography can be incorporated into routine transplantation workflows as an initial outpatient screening modality. The examination is rapid, portable, and relatively low in cost. In candidates with normal CAP values, additional fat-specific imaging may not be required, allowing progression through the standard donor evaluation process without unnecessary delay.

Although overall diagnostic discrimination was comparable between CAP and CT, their performance characteristics differed in clinically meaningful ways. When CAP values were elevated, MRI-PDFF served as a practical confirmatory modality, supporting a stepwise, noninvasive screening approach, particularly in settings with limited MRI availability.

In this proposed stepwise framework, CAP functions primarily as a rule-out tool, while MRI-PDFF provides quantitative confirmation in cases with elevated CAP values. Such a tiered approach may reduce unnecessary MRI utilization while maintaining diagnostic safety. Importantly, given the well-established validation of MRI-PDFF against histology [4], invasive liver biopsy could reasonably be reserved for discordant or high-risk scenarios rather than performed routinely in otherwise healthy donor candidates.

CT demonstrated high specificity and positive predictive value, but lower sensitivity for detecting mild steatosis, consistent with prior reports [9,18,19,20]. While CT remains valuable when MRI is unavailable or when detailed anatomical assessment is required, its limitations in identifying early steatosis should be recognized.

This lack of concordance is not unexpected, as steatosis and fibrosis reflect distinct biological processes that do not necessarily progress in parallel. Previous MASLD (formerly NAFLD) studies have shown that serum fibrosis indices correlate more closely with fibrosis severity than with hepatic fat accumulation [21,22]. Serum fibrosis indices (FIB-4 and APRI) complement imaging by providing biochemical context regarding liver health. Although these markers did not closely mirror imaging-based steatosis findings, they remain valuable components of comprehensive donor assessment, aligning with current MASLD evaluation frameworks that integrate metabolic, structural, and biochemical parameters [23,24].

From a donor safety standpoint, minimizing invasive procedures is especially important in living donor programs, where candidates are otherwise healthy individuals. A predominantly noninvasive strategy may reduce procedural risk, shorten evaluation timelines, and ease donor anxiety, while still maintaining graft safety for recipients. In addition, the stepwise use of CAP followed by selective MRI-PDFF may offer practical cost advantages compared with routine MRI or biopsy-based approaches, although formal economic evaluations are needed to confirm this.

Taken together, these findings extend prior work by demonstrating how commonly used noninvasive tools perform in a healthy, low-prevalence living liver donor population, where diagnostic priorities differ from those in typical MASLD populations. In this screening context, safely excluding clinically significant steatosis is the primary objective. CAP-based transient elastography appears particularly well-suited for disease exclusion in asymptomatic or low-risk individuals, consistent with the prior literature [5,6,14]. These findings also align with donor-specific data reported by Kuru Öz et al. [25], who demonstrated a strong correlation between MRI-PDFF (using both MR spectroscopy and chemical-shift techniques) and histopathology, with a high negative predictive value for excluding clinically significant steatosis in donor candidates.

Conversely, CT maintains high specificity but limited sensitivity for mild steatosis, a trade-off previously reported in healthy subjects and transplant candidates using liver–spleen attenuation differences and transient elastography [9,18,20,26].

PDFF therefore remains the most reproducible noninvasive reference method for hepatic fat quantification and provides an appropriate benchmark for comparative evaluation of screening tools. In contrast, serum fibrosis indices such as FIB-4 and APRI primarily reflect fibrotic risk rather than hepatic fat burden, explaining their limited concordance with imaging-defined steatosis in donor populations [21,22].

**Figure 2:**
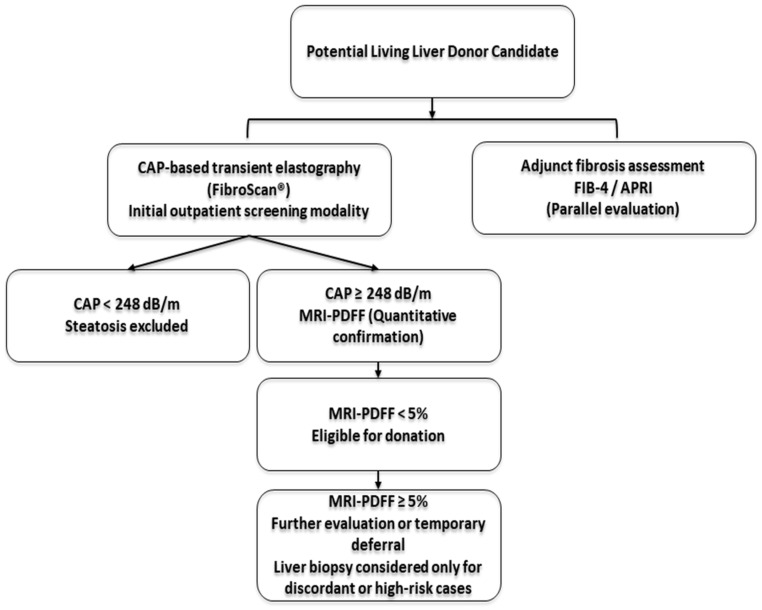
Proposed stepwise noninvasive diagnostic algorithm for living liver donor evaluation integrating CAP-based transient elastography as initial screening and MRI-PDFF as confirmatory assessment when indicated. Invasive biopsy is reserved for discordant or high-risk cases.

Based on the diagnostic performance observed in this cohort, a stepwise noninvasive diagnostic algorithm is proposed for integration into living liver donor evaluation workflows (Figure 2).

### 4.2. Donor Safety and Study Limitations

From a donor safety perspective, reliance on noninvasive tools such as CAP-based transient elastography, CT, FIB-4, and APRI reduces the need for liver biopsy and avoids associated procedural risks—an especially important consideration in living donor programs [12,13].

Several limitations should be acknowledged. This was a single-center, retrospective study, and imaging and laboratory assessments were not always temporally synchronized. As the cohort consisted of healthy donors, findings may not be directly generalizable to individuals with MASLD. Importantly, donor follow-up studies have shown that steatosis and fibrosis progression can develop in the remnant liver years after donation, even in donors initially deemed suitable based on pre-donation imaging, underscoring the importance of accurate baseline assessment and long-term surveillance using noninvasive tools [27].

Histological confirmation of steatosis and fibrosis was not available in this study population, as liver biopsy is not routinely performed in clinically suitable living donor candidates due to its invasive nature and associated risks. Therefore, direct imaging-to-histology concordance could not be assessed within this study. However, MRI-PDFF is well validated and widely accepted as a noninvasive reference method in contexts where biopsy is not clinically justified. MRI-based fat quantification may be influenced by physical factors such as T1 effects (differences in signal recovery between fat and water), T2* effects (signal decay related to magnetic susceptibility and iron content), spectral modeling assumptions, and magnetic field inhomogeneity, whereas histological assessment is not subject to these imaging-related biases [28,29]. In addition, regional heterogeneity of hepatic fat distribution may influence localized measurements, although representative sampling remains common in routine clinical donor evaluation workflows [30].

The sample size was adequate for primary analyses but limited more detailed subgroup comparisons. Despite these limitations, results across modalities were generally consistent and help address an existing gap by characterizing the comparative behavior of noninvasive steatosis assessment tools in a low-prevalence, clinically healthy donor population, where the primary objective is the safe exclusion of steatosis rather than disease staging. Future multicenter studies incorporating histological validation would be valuable for refining diagnostic thresholds, strengthening imaging–histology concordance, and improving generalizability.

## 5. Conclusions

CAP-based transient elastography outperformed CT in identifying MRI-defined hepatic steatosis and functioned well as an initial noninvasive screening tool in living donor evaluation. Its high negative predictive value supports its role as a rule-out modality, potentially limiting unnecessary additional imaging when results are normal. CT remained a valuable confirmatory option when MRI was unavailable. Serum fibrosis indices, particularly FIB-4 and APRI, provided additional biochemical context on liver health and supported a broader assessment of donor suitability.

In the low-prevalence setting of living liver donor evaluation, these findings support a stepwise, noninvasive screening strategy in which CAP-based transient elastography serves as the initial rule-out tool and MRI-PDFF provides confirmatory assessment when indicated. Together, these noninvasive methods offer a practical, low-risk framework that aligns with current MASLD evaluation guidelines. Larger multicenter studies with histological verification are needed to validate these findings and refine diagnostic thresholds for wider clinical use.

## Figures and Tables

**Table 1:** Diagnostic accuracy of CT and FibroScan (CAP ≥ 248 dB/m) for hepatic steatosis compared to MRI (reference standard).

Metric	CT % (95% CI)	FibroScan % (95% CI)
Sensitivity	61.9 (38.1–81.9)	90.5 (69.6–98.8)
Specificity	97.7 (91.9–99.6)	78.2 (68.1–86.0)
PPV	86.7 (62.1–96.3)	50.0 (34.2–65.8)
NPV	91.4 (83.7–96.0)	97.1 (89.9–99.5)
AUC	0.798 (0.667–0.929)	0.832 (0.740–0.924)

Abbreviations: PPV = Positive Predictive Value; NPV = Negative Predictive Value; AUC = Area Under the Curve.

**Table 2:** Confusion matrices for CT and FibroScan (CAP ≥ 248 dB/m) compared with MRI (reference standard).

(a) CT vs. MRI:			
	MRI: Steatosis Present	MRI: No Steatosis	Total
CT: Steatosis Present	13	2	15
CT: No Steatosis	8	85	93
Total	21	87	108
**(b) CAP-Based Transient Elastography (CAP ≥ 248 dB/m) vs. MRI**			
	**MRI: Steatosis Present**	**MRI: No Steatosis**	**Total**
CAP ≥ 248 dB/m (Present)	19	19	38
CAP < 248 dB/m (Absent)	2	68	70
Total	21	87	108

(a) Performance metrics (vs MRI reference):• Sensitivity: 13/(13 + 8) = 61.9%. • Specificity: 85/(85 + 2) = 97.7%. • PPV: 13/(13 + 2) = 86.7%. • NPV: 85/(85 + 8) = 91.4%. (b) Performance metrics (vs MRI reference). • Sensitivity: 19/(19 + 2) = 90.5%. • Specificity: 68/(68 + 19) = 78.2%. • PPV: 19/(19 + 19) = 50.0%. • NPV: 68/(68 + 2) = 97.1%.

**Table 3:** Distribution of fibrosis indices (FIB-4 and APRI) among 108 donors.

Category	FIB-4 (*n*, %)	APRI (*n*, %)
Low risk	102 (96.2%)	103 (97.2%)
Intermediate	3 (2.8%)	2 (1.9%)
High risk	1 (0.9%)	1 (0.9%)
Mean ± SD	0.65 ± 0.60	0.2 ± 0.17
Median (range)	0.52 (0.12–6.04)	0.16 (0.06–1.71)

Interpretation thresholds: FIB-4: <1.3 (low), 1.3–2.67 (indeterminate), >2.67 (high). APRI: <0.5 (low), 0.5–1.5 (indeterminate), >1.5 (high).

## Data Availability

The raw data supporting the conclusions of this article will be made available by the corresponding author upon reasonable request, subject to institutional and ethical restrictions.
